# Study Protocol on Antimicrobial Stewardship in a Tertiary Respiratory Referral Hospital

**Published:** 2018-03

**Authors:** Hamidreza Jamaati, Farzaneh Dastan, Zahra Mirshafiei Langari, Roodabeh Haghgoo, Raha Eskandari, Majid Marjani, Afshin Moniri, Seyed Mohammadreza Hashemian, Behrooz Farzanegan, Atefeh Abedini, Payam Tabarsi, Ali Akbar Velayati

**Affiliations:** 1 Chronic Respiratory Diseases Research Center, National Research Institute of Tuberculosis and Lung Diseases (NRITLD), Shahid Beheshti University of Medical Sciences, Tehran, Iran; 2 Department of Clinical Pharmacy, Faculty of Pharmacy, Shahid Beheshti University of Medical Sciences, Tehran, Iran; 3 Department of Pharmaceutical Care, NRITLD, Shahid Beheshti University of Medical Sciences, Tehran, Iran; 4 Clinical Tuberculosis and Epidemiology Research Center, NIRTLD, Shahid Beheshti University of Medical Sciences, Tehran, Iran; 5 Tracheal Diseases Research Center, NIRTLD, Shahid Beheshti University of Medical Sciences, Tehran, Iran

**Keywords:** Antimicrobial stewardship, Appropriateness, Resistance

## Abstract

**Background:**

Antimicrobial stewardship program is a comprehensive, longitudinal program designed to improve and measure the appropriateness of antimicrobial use while increasing patients' safety, decreasing cost of patients' care, and combating emerging antimicrobial resistance. Antimicrobial resistance, specially emerging multidrug-resistance and extremely drug-resistance gram negative bacteria, is an important concern in the modern world. This is particularly problematic since antimicrobials in production pipelines are not meeting the demand for the emerging resistance micro-organisms; in another word “we are running out of options”. Indiscriminate use of antimicrobial may increase the risk for resistance, and drug toxicity. The aim of this study is to implement an evidence-based antimicrobial stewardship program in a tertiary referral hospital. This study will assure consistency of the stewardship program and measure outcomes to further assess the effectiveness of this program.

**Materials and Methods:**

After establishment of antimicrobial stewardship committee and endorsement of policies the program will be conducted in all hospital medical wards. In an observational study, all patients receiving antimicrobials included in the program will be closely monitored for primary and secondary outcomes. Hospital's antimicrobial resistance patterns are monitored periodically to assess improvement. The quality indicators will be assessed to ensure proper execution of the program over time.

**Results:**

As a study protocol, there are no results available to be reported at this time.

**Conclusion:**

We are expecting to observe significant reduction in cost of antibiotic use shortly after program execution. By more appropriate utilization of antibiotics patients' safety will be increased. Furthermore, we are expecting to detect improvement in antimicrobial resistance patterns.

## INTRODUCTION

Since early 20^th^ century, discovery of antibiotics have changed the practice of medicine, in a sense that once lethal infections, are now readily treatable. On one hand increased risk of morbidity and mortality is associated with inadequate empiric therapy in critically ill patients and on the other hand indiscriminate use of antibiotics in these patients promotes generation of resistance that can affect the whole population (1). By some estimates, up to 50% of antimicrobial drugs are used unnecessarily or inappropriately (2). Emergence of resistance, toxicity, prolonged hospital length of stay, increased morbidity and mortality, increased cost of care, *Clostridium difficile* (*C. difficile*) and other Hospital–Acquired Infections (HAI), are all potential aftermaths of inappropriate use of antimicrobials (1,3).

Resistance to antimicrobials is a growing threat to public health because specific treatment options are limited for the emerging multidrug and extremely drug resistant species (4). Moreover, analysis of antibiotics development pipeline raises a serious concern on lack of new antibiotics of emerging resistant organisms (5).

ESCAPE-*Enterococcus faecium, Staphylococcus aureus, C. difficile, Acintobacter baumanni, Pseudomonas aeruginosa,* and *Enterobacteriaaceae* [which includes *Enterobacter* species, *Klebsiella pneumoniae* (*K. pneumoniae), Klebsiella oxytoca, Escherichia coli (E. coli),* and *Proteus mirabilis*]-formerly known as ESKAPE, with K for *K. pneumonia* and E for *Enterobacter* species, are the most troublesome since they often confer resistant to more than one agent when causing HAI (6,7).

Antimicrobial Stewardship Program (ASP) has been defined by Infectious Diseases Society of America (IDSA), Society for Healthcare Epidemiology of America (SHEA), and Pediatrics Infectious Disease Society (PIDS) as “coordinated interventions designed to improve and measure the appropriate use of antibiotic agents by promoting the selection of the optimal antibiotic drug regimen including dosing, duration of therapy, and route of administration” (8). The primary goals of ASP is to confront unintended consequences of antimicrobial use, including emergence of resistance, and optimize clinical outcome (2). This program will help to improve quality of patient care and ameliorate patients safety through raising infection cure rates, and reducing treatment failures (9,10).

Secondary goal of ASP is to reduce cost of care without compromising quality of care. In recognition of the need for an improvement in antibiotic use in hospitals, Center of Disease Control and Prevention (CDC) has recommended all acute care hospitals to implement Antimicrobial Stewardship Program (2). Comprehensive programs have demonstrated to be financially self-supporting since implementation of ASP can lead to 22–36% reduction in antimicrobial use, and consequent annual cost saving of $200,000–$900,000 (11–13).

This ASP will be initiated in a respiratory tertiary referral hospital, aiming to optimize antimicrobial use, combat antimicrobial resistance, and increase quality of care while decreasing cost of care.

## MATERIALS AND METHODS

### A) Antimicrobial Stewardship Policy:

Hospital shall develop its local policy. Antimicrobial Stewardship Committee (ASC) must be established and all stewardship policies must be endorsed by the committee. Policies must then be publicized to the whole facility by hospital director. The policy shall include the following:

1. Ratification of a list of antimicrobial agents that must be included in the ASP. Primarily broad spectrum antibiotics will be included in the program, which includes: vancomycin, meropenem, imipenem, aminoglycosides (amikacin, gentamycin, tobramycin), fluoroquinolones (ciprofloxacin, levofloxacin, ofloxacin, moxifloxacin) colistin, linezolid, amphotericin B, caspofungin, and voriconazole. All available forms of above mentioned drugs will be included in the study.
2. Indications for antimicrobials are to be explicitly spelt out at the time of prescribing.
3. Two blood cultures must be performed before antimicrobial initiation.
4. Cultures must be performed from suspected sites of infection.
5. Infection control measures including proper hand hygiene must be performed.

### B) Core Strategies:

#### I) Antimicrobial Stewardship Team and Leadership:

Core members of a multidisciplinary antimicrobial stewardship team will include an infectious disease physician and a clinical pharmacist with infectious disease training, and a clinical microbiologist. The ASP team will further include an information system specialist (14). The program will be codirected by the infectious disease specialist and clinical pharmacist. They will obtain adequate authority from hospital administration.

A single physician will be appointed as ASP leader and must be responsible for the program outcome. Leadership support is critical in a successful antimicrobial stewardship program, and will take a number of forms; policies made by the stewardship committee will be affirmed by the leader. Furthermore, support of hospital administration and other key groups in the hospital greatly enhance implementation of ASP. Support of clinicians and nurses will be drawn by involving key person of each group in the ASC (14).

#### II) Interventions:

##### 1. Formulary restriction and preauthorization

This method can result in immediate and significant reduction in antimicrobial cost (15,16). Formulary restriction and preauthorization limit the use of certain antimicrobials with very specific indications and spectrum of activity (ex: Linezolid, Antifungals) and require the clinician to obtain approval of clinical pharmacist and infectious disease attending. Drug culture and antimicrobial susceptibility tests must be available at the time of approval. Using computerized decision making system, pharmacy will be informed of antimicrobial prescriptions (17).

Overall trend of antimicrobial use must be monitored periodically since in this method antimicrobial utilization may shift to alternative agents (2).

##### 2. Prospective audit with intervention and feedback

Prospective Audit and Feedback (PAF) with direct interaction of an infectious disease physician by medical consult and giving direct feedback to the prescriber will be used to increase compliance to local guidelines (17).

### C) Supplemental Strategies:

#### I) Implementation of facility-specific clinical practice guideline:

Facility-specific treatment recommendations will be developed and implemented based on national guidelines and local susceptibility for common infectious disease syndromes. Local antimicrobial susceptibility pattern will be considered when initiating empiric therapy for specific infections. Local guidelines will be endorsed by ASC and be announced by ASP leader to hospital clinicians (18).

#### II) Antimicrobial “Time out”:

Implementation of antimicrobial “time out” or stop order will further oblige clinicians to revise antimicrobial agents after 72-hours of initiation. Computerized decision support at the time of prescribing will be incorporated as a part of ASP to facilitate time-sensitive stop order implementation (14).

#### III) Pharmacy-driven interventions:

Clinical pharmacist consult to perform dose adjustments for patients with organ dysfunction in order to optimize the dose and frequency of drug therapy will be performed (18).

#### IV) Educational interventions:

Educational interventions will be incorporated into ASP through passive activities like conference presentations for hospital personnel and clinicians (19).

### D) Strategies for Optimization of ASP:

I) Pharmacist-driven pharmacokinetic drug monitoring and dose optimization will be implemented as a part of ASP program for aminoglycosides and vancomycin (18).
II) Guideline for appropriate initiation of oral antibiotics and timely transition of patients from IV to oral antibiotics will be designed and implemented in hospital (20).
III) De-escalation of therapy by implementation of intervention to reduce antimicrobial therapy to the shortest effective duration will be performed. Automatic antibiotic time-out through computerized decision making system can aid this intervention. Stop orders will be implemented at day 7 and 14 of antimicrobial therapy and maximum allowance time for drug therapy will be defined and can be applied through pharmacy ordering system (20).

Discontinuation of inappropriate or redundant antimicrobial therapy based on culture and antimicrobial susceptibility data is another approach to de-escalate antimicrobial therapy. Broad-spectrum antimicrobial therapy will be discontinued and targeted therapy should be initiated (2). However, since clinicians are reluctant to changing or stop therapy in cases of negative cultures and clinical improvement, taking any decision in such situation will depend on clinical judgment (21).

### E) Microbiology and Laboratory Diagnosis:

I) ASP will work with the microbiology laboratory to develop stratified antimicrobial susceptibility tests according to the latest Clinical and Laboratory Standard Institute (CLSI) guidelines.
II) ASP will work with the microbiology laboratory to preform selected or cascade reporting of antibiotic susceptibility test results.
III) Use of serial PCT for adult ICU patients with suspected infection must be recommended by ASP (18).

### F) Antimicrobial Measurements:

I) Defined Daily Dose (DDD), Day of Therapy (DOT), and Length of Therapy (LOT) are used as measures to monitor antimicrobial therapy.
II) Evaluation of antimicrobial cost will be performed based on prescription or administration to monitor hospital expenditures (18).

### G) Process and Outcome Measures:

Excess days of therapy, duration of therapy, proportion of patients receiving therapy as per facility guideline or algorithm, will be assessed and ASP should be revised based on these data. Microbiology guideline, and proportion of patients converted to oral therapy can be used as measures to assess process of ASP.

Furthermore, hospital length of stay, 30-day mortality, unplanned hospital readmission within 30 days, proportions of patients with clinical failure (eg, need for broad spectrum antimicrobial, recurrence of infection), proportion of patients diagnosed with *C. difficile* or adverse events related to antibiotic treatment, and also the percent of antibiotic resistant healthcare-associated pathogens prevalence will be employed to measure ASP outcomes.

## DISCUSSION

Our antimicrobial stewardship program will be implemented with the aim to improve hospital's quality of patient care; while combating the bacterial resistance which is a major concern in the healthcare system (8). ASPs are dedicated to improve antimicrobial use both through optimization of treatment and by reducing antimicrobial related adverse events (9). These programs can be utilized to further improve patient safety by reducing treatment failure, increasing rationalized use of antibiotics and cure rates (10–22).

Longitudinal study of ASP outcome measures and quality indicators will guarantee sustainability and effectiveness of the program.
